# Abnormal Thyroid Function Laboratory Results Caused by Selective Serotonin Reuptake Inhibitor (SSRI) Antidepressant Treatment

**DOI:** 10.1155/2023/7170564

**Published:** 2023-05-11

**Authors:** Huijuan Liao, David S. Rosenthal, Salini C. Kumar

**Affiliations:** Division of Endocrinology, Nassau University Medical Center, 2201 Hempstead Turnpike, East Meadow, NY 11554, USA

## Abstract

Mental health issues, especially depressive disorders, are major burdens to the health care systems. This has been more pronounced since the onset of the COVID-19 pandemic. Selective serotonin reuptake inhibitors (SSRIs) are often prescribed for depression. Uncommonly appreciated, however, are the adverse effects these agents may have on thyroid function laboratory test results as well as the clinical thyroidal functional status of such patients, which may lead to erroneous diagnoses and inappropriate treatments. We report on a depressed woman who developed abnormal thyroid biochemical laboratory reports during fluoxetine therapy. After changing to the serotonin and norepinephrine reuptake inhibitor (SNRI) venlafaxine, the thyroid laboratory reports were normalized. In light of this, we wish to alert treating clinicians to this potential significant adverse effect.

## 1. Introduction

Mental health problems, especially depressive disorders resulting in disability, lead to significant personal, societal, and monetary costs—estimated in America at $326 billion in 2018 [1]. Reports suggest that 10% of all adults suffer from significant depression each year with a lifetime prevalence of 20.6% [2], which accounted for 15.9% of visits to primary care physicians in 2018 [3]. This has been exacerbated by the COVID-19 pandemic [4]. Furthermore, adults over the age of 65 years often present a higher therapeutic challenge due to comorbidities [5].

A recommended first-line treatment for acute mild depressive disorder is cognitive behavioral therapy with, if needed, the addition of a second-generation antidepressant, such as fluoxetine. An initial treatment recommendation for acute moderate to severe depressive disorder is cognitive behavioral therapy, a second-generation antidepressant alone or the combination, as required by the individual patient [6, 7]. Selective serotonin reuptake inhibitors (SSRIs) are often chosen as the initial antidepressant medication. Uncommonly appreciated, however, are the adverse effects these agents may have on thyroid function laboratory test results as well as on the clinical thyroidal functional status resulting from an SSRI alteration of thyroid follicular function [8].

Here, we report the case of a patient whose thyroid laboratory test results became abnormal when SSRI therapy was initiated. The abnormality was resolved when the medication was changed to a serotonin and norepinephrine reuptake inhibitor (SNRI).

## 2. Case Report

A 70-year-old clinically euthyroid woman without any past history of thyroid disease had normal thyroid biochemistry results when seen by her primary care physician on December 26, 2020: thyroid stimulating hormone (TSH) 0.428uIU/ml (reference values 0.282-4.0) and total triiodothyronine (TT3) 94 ng/dL (72-180) (Table 1). Six months later, the patient was prescribed fluoxetine 20 mg daily because of a new diagnosis of depression. The treatment continued when the patient was transferred to a skilled nursing center four weeks later. At that time, TSH was suppressed (0.04uIU/mL [0.55-4.78]), although she continued to be clinically euthyroid.

When an enlarged thyroid gland was later suspected and repeated TSH testing was unchanged, the patient was referred to our facility for endocrine evaluation, where she was seen on April 14, 2022. We found her vital signs to be: blood pressure 145/79 mm Hg, heart rate 93/minute, respiratory rate 18/minute. She reported having had a benign thyroid nodule, which was resected ten years previously. Additional medical history included type 2 diabetes mellitus, hyperlipidemia, and mild residual right-sided weakness from a cerebral vascular accident one year earlier. The patient was felt to be clinically euthyroid although with a slight right-sided tremor attributed to her stroke history. In addition to fluoxetine 20 mg daily, her medications included metformin 500 mg twice daily and atorvastatin 80 mg daily. The patient was confirmed to have a slightly enlarged thyroid gland (right lobe 6.3 × 3.5 × 2.3 cm; left lobe 6.8 × 3.3 × 2.5 cm) with bilateral nodularity. Fine needle biopsies reported all nodules to be benign (Bethesda category 2). The 24-hour radioiodine uptake study was normal at 29.5 (8-35)% with a slightly heterogeneous pattern but without photodense or photopenic areas on the isotope scan. TSH was suppressed at 0.07 uIU/mL (0.55-4.78), while total thyroxine (TT4), free thyroxine (fT4), TT3, and thyroid-stimulating immunoglobulin (TSI) were all within normal limits (Table 1). Antithyroperoxidase (antiTPO) and antithyroglobulin (antiTG) antibodies were undetectable.

Because of the suspicion that fluoxetine was causing the TSH suppression, treatment was changed two months later (August 29, 2022) to the SNRI venlafaxine 37.5 mg daily. Once SSRI medication was discontinued, TSH improved back to the patient's prior baseline levels at 0.63, 0.46, and 0.42 uIU/mL, and the patient remained clinically euthyroid (Table 1).

## 3. Discussion

We report a case of a clinically euthyroid woman with normal thyroid biochemistry who developed suppression of her TSH laboratory result when treated with the SSRI fluoxetine for depression. The abnormal TSH laboratory results resolved back to her baseline levels when treatment was changed to the SNRI agent venlafaxine.

The results are partly in agreement with two previous case reports who found biochemical and clinical hyperthyroidism after treatment with fluoxetine [9, 10]. However, in contrast to these previous reports, our patient had reduced TSH levels only and no other laboratory or clinical signs of hyperthyroidism. Four other studies reported cases with biochemical and clinical hypothyroidism after treatment with the SSRIs escitalopram [11, 12], paroxetine [13], and sertraline [14].

In a case series, König et al. [15] noted the stability of TSH and decrease in TT4 and fT4, and inconsistently, both elevated and reduced TT3 in patients taking the SSRI paroxetine. Joffe and Singer [16] reported decreases in TT4 and fT3 with TSH unchanged in patients using tricyclic antidepressants. Shelton et al. [17] documented decreases in T3 with fluoxetine.

While poorly understood, it has been suggested that thyroid follicular cells respond to the neurotransmitter serotonin (5-hydroxytryptamine (5-HT)) and, hence, may be affected by SSRIs [18]. In vitro studies have found decreased storage and secretion of thyrotropin-releasing hormone (TRH) in cultured fetal rat hypothalamic neurons exposed to SSRIs, thereby having an inhibitory effect on pituitary TSH release [19].

The strength of this study is the clear temporal relationship between the prescription of fluoxetine and low TSH levels. A limitation is that we did not have any serum concentrations of fluoxetine. However, it was administered under supervision at the nursing home.

## 4. Conclusion

Variable but infrequent effects of SSRI treatment of depression upon thyroidal physiology and laboratory reports, as well as clinically significant hypo and hyperthyroidism, have been reported. It is important to bring this issue to the attention of treating and consulting physicians to avoid misdiagnosis and erroneous treatments.

## Figures and Tables

**Table 1:** Summary of thyroid biochemical results.

	Dec. 26, 2020	July 2, 2021	April 5, 2022	April 15, 2022	Sept. 22, 2022	Oct. 24, 2022	Nov. 24, 2022
TSH (0.55-4.78 uIU/mL)		0.04	0.04	0.07	0.63	0.46	0.42
TSH (0.282-4.0 uIU/mL)	0.482						
TT4 (3.2-1236 *u*g/dL)				8.8	8.4	7.3	8.7
fT4 (0.8-1.8 ng/dL)				1.1	1.1	1.1	1.2
TT3 (76-181 ng/dL)	94			81	87	82	76
fT3 (2.3-4.2 pg/L)						2.9	2.9
TSI (<140%)				<89	<89	<89	<89
AntiTPO (<9 IU/mL)				<1	<1		<1
AntiTG (<1 IU/mL)				<1	<1		<1
Antidepressant pharmacotherapy	None	←------------------------------------------------→			←--------------------------------------------------→		
		Fluoxetine 20 mg/d			Venlafaxine 37.5 mg/d		

Abbreviations: TSH: thyroid stimulating hormone; TT4: total thyroxine; fT4: free thyroxine; TT3: total triiodothyronine; fT3: free triiodothyronine; TSI: thyroid stimulating immunoglobulin; AntiTPO: antithyroperoxidase; AntiTG: antithyroglobulin.

## Data Availability

The data used to support the findings of this study are included within the article and the cited references.
